# Same tumor, different lungs: How chronic obstructive pulmonary disease and interstitial lung disease reshape lung cancer risk and treatment

**DOI:** 10.1515/jtim-2026-0059

**Published:** 2026-07-21

**Authors:** Xipei Song, Xiangyu Zhang, Qibin Lin, Xinyu Jin, Molly Li, Xiuning Le, Yang Xia

**Affiliations:** Department of Respiratory and Critical Care Medicine, Key Laboratory of Respiratory Disease of Zhejiang Province, Second Affiliated Hospital, Zhejiang University School of Medicine, Zhejiang University, Hangzhou, Zhejiang Province, China; State Key Laboratory of Translational Oncology, Department of Clinical Oncology, The Chinese University of Hong Kong, Hong Kong, China; Department of Thoracic/Head and Neck Medical Oncology, University of Texas MD Anderson Cancer Center, Houston, Texas, USA

Lung cancer (LC) is the deadliest form of cancer worldwide and ranks as the seventh leading cause of death globally. Chronic obstructive pulmonary disease (COPD) and interstitial lung disease (ILD) are common respiratory diseases with poor prognosis. Many studies have found that both conditions frequently co-occur with LC in the same patient, known as LC-COPD and LC-ILD. The presence of COPD or ILD significantly alters the pathogenesis, diagnosis and treatment for lung cancer, making its management different from that of lung cancer alone and necessitating comprehensive considerations in clinical decision-making (Figure 1). As a result, they have become increasingly prominent medical issues.

**Figure 1:**
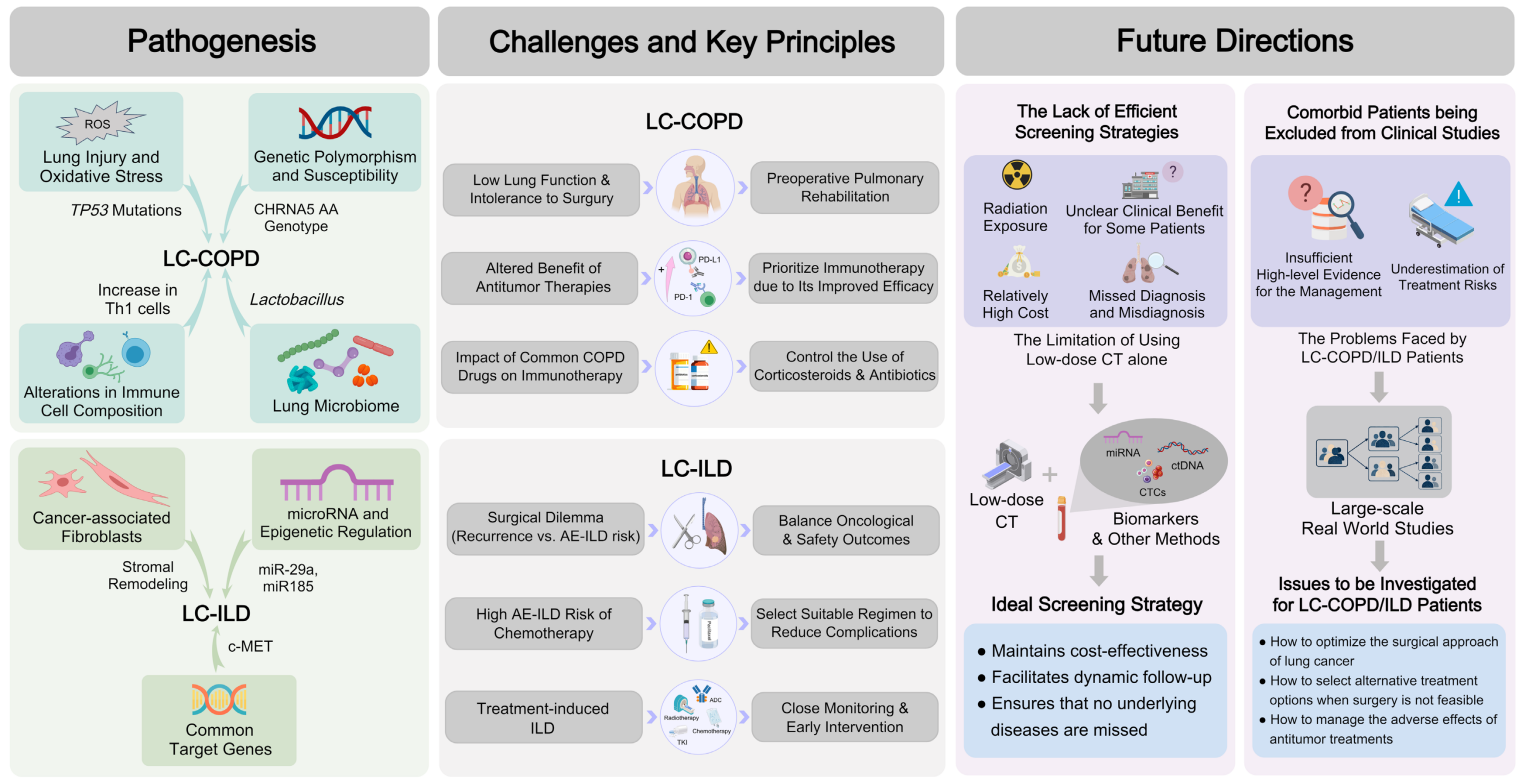
Overview of the pathogenesis, clinical challenges with key therapeutic principles and future directions for LC-COPD/ILD. LC: lung cancer; COPD: chronic obstructive pulmonary disease; ILD: interstitial lung disease.

## Epidemiology of LC-COPD and LC-ILD

COPD has been well-recognized as a risk factor for lung cancer. Never smokers with COPD have more than 2.6 times the incidence of lung cancer compared to those without COPD.^[1]^ The incidence density of lung cancer among COPD patients is 16.7 per 1000 person-years, with squamous cell carcinoma being the most common histological type, followed by adenocarcinoma and small cell lung cancer (SCLC).^[2]^ Therefore, the risk of lung cancer in COPD patients should be given high priority.

ILD is a group of highly heterogeneous diseases, including over 200 subtypes. Increased prevalence of lung cancer has been reported in many ILD subtypes, among which idiopathic pulmonary fibrosis (IPF) is most frequently observed. The prevalence of lung cancer in IPF patients can reach 15.3%, where squamous cell carcinoma accounts for the highest incidence, ahead of adenocarcinoma and SCLC, and the survival rate of LC-IPF patients is significantly lower than that of IPF patients alone.^[3]^ Moreover, the cumulative incidence of lung cancer in IPF patients increases progressively over time, with reported incidence rates of 1.1%, 15.9%, and 31.1% at 1-, 5-, and 10-years post-diagnosis, respectively.^[3]^ Consequently, effective lung cancer screening and early treatment for ILD patients are crucial clinical strategies for improving outcomes.

## Pathogenesis of LC-COPD and LC-ILD

Both LC-COPD and LC-ILD share common risk factors and pathogenic pathways. A central mechanism is chronic inflammation, which promotes cellular damage, genomic instability, and a tumor-promoting microenvironment. In COPD, it originates from oxidative stress caused by factors such as smoking, air pollution, and occupational dust exposure; in ILD, it originates from damage and abnormal repair of alveolar epithelial cells. In addition, aging is also a common risk factor for these diseases.

The biological basis for the close association between COPD and lung cancer lies in many aspects. A recent study has shown that COPD reshapes the tumor immune microenvironment in non-small-cell lung cancer (NSCLC). Specifically, patients with COPD exhibit an expansion of Th1 cell populations and an increase in Interferongamma (IFN-γ) producing CD8^+^ T cells in both lung parenchyma and matched tumor tissue. Furthermore, the presence of COPD is associated with enhanced expression of immune checkpoints like PD-1 in tumors.^[4]^ Besides, genetic susceptibility (such as CHRNA AA genotype)^[5]^ and alterations in the lung microbiome (such as an increase in *Lactobacillus*)^[6]^ have both been shown in recent years to be associated with the occurrence and progression of lung cancer and COPD.

The pathogenesis of lung cancer and ILD also shares closely related biological mechanisms. A recent study reveals that lung adenocarcinoma occurring in the setting of ILD possesses an altered cancer-associated fibroblast (CAF) phenotype, which is characterized by significantly higher expression of some markers and elevated presence of novel matrix-related proteins.^[7]^ These findings indicate that the chronic injury and remodeling processes of ILD create a unique fibro-inflammatory microenvironment that likely contributes to the development of lung cancer. Additionally, epigenetic regulation via microRNA expression and mutations of certain proto-oncogenes and tumor suppressor genes in IPF patients, have both been proved to play important roles in the pathogenesis of LC-ILD.^[3]^

## Management of LC-COPD and LC-ILD

Patients with LC-COPD/ILD represent a distinct population requiring highly individualized attention. Their clinical condition frequently presents challenges such as conflicting treatment goals and cumulative risks. Therefore, clinical decision-making should be guided by both existing medical evidence and a treatment plan formulated by the multidisciplinary team (MDT).

Several key points warrant attention in the management of LC-COPD patients. First, both diseases need to be treated concurrently. For LC-COPD patients with high surgical risk, preoperative COPD treatment and pulmonary rehabilitation are recommended to be initiated first. When selecting a chemotherapy regimen, the patient’s GOLD grade may also be considered. Second, immunotherapy represents a priority treatment option. Numerous recent studies have confirmed that COPD is an independent predictive factor for improved outcomes in NSCLC patients receiving immunotherapy,^[4]^ which may be attributed to its impact on the tumor immune microenvironment. Third, it is crucial to monitor drug interactions closely. For example, studies have found that high-dose systemic corticosteroids and antibiotics may diminish the efficacy of immunotherapy.^[8,9]^ Given that LC-COPD patients often present with complex conditions and are likely to require both types of medications, their indications and dosages need careful management in clinical practice.

As for LC-ILD patients, multiple factors including efficacy and safety need to be comprehensively considered. First, when selecting surgical procedures, consideration requires not only to reducing cancer recurrence rates but also to minimizing the risk of perioperative acute exacerbation of ILD (AE-ILD). Studies indicate that wedge resection and segmentectomy carry a lower risk of AE-ILD but higher cancer recurrence rates compared to lobectomy.^[3]^ Therefore, lobectomy is recommended whenever possible, except for patients at high risk of AE-ILD. Second, medication regimens necessitate cautious selection to avoid inducing AE-ILD. Pharmacological treatments may cause AE-ILD in 5%–20% of LC-ILD patients, with a mortality rate as high as 30%–50%.^[10]^ Different chemotherapy regimens vary in their efficacy and risk of AE-ILD, so the choice should be based on the patient’s specific condition and current evidence. Third, the timing and modality of radiotherapy call for careful deliberation. Studies have shown that radiotherapy significantly increases the incidence of radiation pneumonitis and reduces survival in LC-ILD patients, even with high-precision techniques such as stereotactic body radiotherapy (SBRT).^[3]^

## Future directions

Patients with COPD/ILD have an increased risk of lung cancer, which highlights the importance of screening. However, currently used low-dose CT presents challenges such as high costs, elevated false-positive rates, and limitations for short-term follow-up. Besides, CT screening shows little benefit in advanced COPD (GOLD 3–4),^[11]^ and lung cancers in IPF patients are often obscured by fibrotic changes on imaging, leading to missed or misdiagnosed cases. Therefore, there is a need to explore novel screening methods suitable for patients with lung cancer comorbidities. Biomarker-based screening approaches, such as miRNA, ctDNA and circulating tumor cells, offer a non-invasive, convenient, and cost-effective alternative with good sensitivity and specificity, making them suitable for potential lung cancer patients with COPD/ILD. Furthermore, artificial intelligence (AI) can assist in the clinical management of such patients. AI models enable more precise diagnose and risk stratification by integrating multimodal data, including radiomic features and liquid biopsy biomarkers. This approach also supports personalized treatment planning and improves the prediction of disease progression.

Patients with comorbidities are frequently excluded from clinical research, resulting in a scarcity of high-level medical evidence for this population. Therefore, researchers should conduct large-scale real-world studies specifically targeting comorbid patients, and actively explore innovative therapies that address both tumor control and the handling of underlying lung diseases. The role of traditional Chinese medicine (TCM) should be noted here. For example, dihydroquercetin, an active component in TCM, inhibits ferroptosis in COPD and pulmonary fibrosis by activating specific signaling pathways and regulating ferritinophagy, thereby alleviating lung injury in both diseases. This compound also exhibits potential antitumor activity.^[12]^ Future research needs to focus on several questions about the management of LC-COPD/ILD, including the optimization of surgical techniques, the selection of alternative treatment options when surgery is not feasible, and strategies to mitigate treatment-related adverse effects.

Many connections between lung cancer and COPD/ILD still remain unclear. Deeper insights into these connections will facilitate the development of novel therapies. For instance, COPD has diverse subphenotypes with various immune profiles. How this heterogeneity impacts the onset and progression of lung cancer may point the way for the improvement of immunotherapy. Concomitantly, understanding how ILD affects CAFs at genetic and molecular levels can guide microenvironment-targeted strategies.
